# Effects of DHA Oral Supplementation on Plasma Resolvin D1 and D2 Levels in Naïve Breast Cancer Patients

**DOI:** 10.3390/cancers17101694

**Published:** 2025-05-18

**Authors:** Alessio Molfino, Giovanni Imbimbo, Gerardo Salerno, Luana Lionetto, Alessandro De Luca, Maurizio Simmaco, Carmen Gallicchio, Orietta Picconi, Maria Ida Amabile, Maurizio Muscaritoli

**Affiliations:** 1 Department of Translational and Precision Medicine, Sapienza University of Rome, 00185 Rome, Italy; giovanni.imbimbo@uniroma1.it (G.I.); carmen.gallicchio@uniroma1.it (C.G.); maurizio.muscaritoli@uniroma1.it (M.M.); 2 Analytical Laboratory Unit, Department NESMOS, Sant’Andrea Hospital, Sapienza University of Rome, 00185 Rome, Italy; gerardo.salerno@uniroma1.it (G.S.); luanalionetto@gmail.com (L.L.); maurizio.simmaco@uniroma1.it (M.S.); 3 Department of Surgical Sciences, Sapienza University of Rome, 00185 Rome, Italy; alessandro.deluca@uniroma1.it (A.D.L.); mariaida.amabile@uniroma1.it (M.I.A.); 4 National HIV/AIDS Center, Istituto Superiore Di Sanità, 00161 Rome, Italy; orietta.picconi@iss.it

**Keywords:** specialized pro-resolving lipid mediators, resolvin D1, resolvin D2, breast cancer, docosahexaenoic acid

## Abstract

**Background/Objectives**: Specialized pro-resolving lipid mediators, such as resolvins derived from omega-3 fatty acids, play a key role in resolving inflammation and restoring homeostasis. Resolvin D1 and D2, derived from docosahexaenoic acid (DHA), have demonstrated inflammation pro-resolving properties and potential anticancer effects. This study aimed to evaluate the effects of oral DHA supplementation on plasma resolvin D1 and D2 levels in breast cancer patients and in controls, and by stratifying the patients by disease presentation (sporadic, familial, BRCA1/2 mutated) and immunohistochemical characteristics. **Methods**: This is a single-center, interventional, controlled study conducted in women with breast cancer and women with benign breast disease, serving as controls. Participants consumed DHA (2 g/day) as algal oil syrup for 10 consecutive days. Plasma resolvin D1 and D2 levels were measured at baseline (T0) and after supplementation (T1) using ELISA kits. **Results**: At baseline, breast cancer patients exhibited higher plasma resolvin D1 levels compared to controls (median 21.3 vs. 7.3 pg/mL, *p* = 0.039), with no significant difference in resolvin D2. Following DHA supplementation, resolvin D1 and D2 significantly increased in BRCA1/2-mutated patients (+185.8% and +101.2%, *p* = 0.037, *p* = 0.028, respectively). Conversely, the familial breast cancer group showed a significant decrease in resolvin D1 (*p* = 0.015). Patients with low Ki67 expression showed greater increase over time of resolvin D2 levels compared to those with high Ki67 expression (*p* = 0.046). **Conclusions**: DHA supplementation modulated resolvin levels in breast cancer patients, with significant increase in BRCA1/2-mutated patients, suggesting enhanced inflammation pro-resolving responses. The reduction in resolvin D1 in the familial group highlights a potential dysregulated response. These findings indicate the potential of resolvins as biomarkers of resolution of inflammation and novel therapeutic targets in breast cancer.

## Simple Summary

Breast cancer is often linked to chronic inflammation; however, our capacity to understand and therapeutically target this clinically relevant aspect of the disease remains limited. Specific molecules named resolvins, naturally produced in the body from omega-3 fatty acids like DHA, are involved in resolving systemic inflammation and may have anticancer properties. This study explored whether oral DHA supplementation could change the levels of two types of resolvins—RvD1 and RvD2—in women recently diagnosed with breast cancer. We found that women with BRCA1 or BRCA2 genetic mutations had the most significant increase in these molecules after supplementation, suggesting a stronger anti-inflammatory response. Conversely, women with a family history of breast cancer without a mutation showed a decrease in RvD1. These results suggest that dietary DHA or its supplementation may affect inflammation differently depending on cancer subtype and genetics, and could offer new ways to personalize metabolic and nutritional care in breast cancer.

## 1. Introduction

Breast cancer represents a relevant health burden, affecting over 2.3 million women worldwide in 2022 [1,2]. This condition is characterized by a complex interplay with environmental factors, genetic and epigenetic alterations, as well as with inflammation and immune deregulation [3,4,5,6]. Inflammation has long been recognized as a hallmark of cancer [7], contributing to its initiation, progression, and resistance to therapy [8]. In particular, chronic inflammation may promote tumor development through sustained cytokine production, immune evasion, and angiogenesis [9,10]. Within this framework, specialized pro-resolving lipid mediators (SPMs), including resolvins derived from DHA, act as endogenous brakes on inflammation [11,12].

Recent findings highlighted the role of specialized pro-resolving lipid mediators (SPMs), including resolvins derived from omega-3 fatty acids, in orchestrating the resolution of inflammation and restoring homeostasis in cancer [13].

As described by Serhan et al. [14], the active resolution of acute inflammation has emerged as a critical point of interaction between the innate and adaptive immune systems, and this process involves dynamic biochemical pathways that facilitate the restoration of tissue homeostasis. Data showed the relevant role of eicosanoids such as lipoxins, along with more recently identified classes of endogenous lipid mediators, including resolvins and protectins. These compounds present both anti-inflammatory and pro-resolving activities, contributing to organ protection, the efficient removal of inflammatory by-products, and the reinforcement of mucosal antimicrobial defenses [14].

Among these mediators, resolvins of the D series derived from docosahexaenoic acid (DHA) have demonstrated potential anti-inflammatory and anticancer properties [15,16,17].

Our previous research profiled plasma levels of some resolvins in breast cancer patients and identified significant associations with the type of disease presentation, genetic signature (BRCA1/2 mutation), and immunohistochemical characteristics, including the triple-negative phenotype and high Ki67 expression [18].

These findings underlie the potential role of resolvins as novel biomarkers and therapeutic targets in breast cancer management. Incorporating resolvins into clinical biomarker panels may improve patient stratification and individualized treatment planning, especially for controlling inflammation.

However, the impact of modulating resolvin levels in breast cancer through dietary or pharmacological interventions remains unexplored. In particular, DHA supplementation is able to specifically modulate resolvin D1 and D2, which are bioactive lipid mediators generated from DHA [19].

For this reason, in this study we aimed to evaluate the effect(s) of oral DHA supplementation on plasma resolvin D1 and D2 levels in BC patients compared to controls and to verify differences in their concentrations according to the type of disease presentation (sporadic, familial, or BRCA1/2-mutated) and immunohistochemical characteristics.

## 2. Materials and Methods

### 2.1. Study Design

This was a single-center, interventional, controlled study. Participants were recruited from patients diagnosed with primary breast cancer or with benign breast disease (controls) at the Department of Surgical Sciences, Sapienza-University of Rome, Italy, and patients were naïve to any anticancer medical or surgical treatments. Inclusion criteria were: women aged ≥18 years, histologically confirmed breast cancer or benign disease for controls, and the ability to provide informed consent. Patients with known acute or chronic inflammatory diseases or recent use of omega-3 supplements (within the last 6 months) were excluded. The study protocol was approved by the local Ethics Committee (prot no. 588/13), and all participants provided written informed consent.

### 2.2. Intervention: DHA Oral Supplementation

DHA was administered to patients and controls in the form of algal oil syrup derived from *Schizochytrium* sp. containing 10% DHA (strawberry-flavored Richoil^®^ syrup, DMF, Italy), as previously described [20]. The product was provided free of charge by the manufacturer. Each participant consumed 10 mL of the syrup twice daily for 10 consecutive days, providing a total daily DHA intake of 2 g. The choice of a 10-day intervention period was based on our previous studies using the same DHA formulation and dosage [20,21]. In fact, a 10-day supplementation was sufficient to significantly increase DHA levels in red blood cell membranes and to significantly elevate the circulating levels of 19,20-EDP, which is a bioactive DHA-derived lipid mediator [20,21].

During the intervention period, participants were instructed to follow a standard normo-balanced diet and maintain their usual physical activity levels. As previously implemented [20], a dedicated contact number was provided to ensure compliance and address any issues or concerns during the study.

### 2.3. Sample Collection and Measurement of Plasma Resolvin D1 and D2

Blood samples were collected at baseline (before DHA supplementation) (T0) and at the end of the intervention period (T1). Samples were drawn in the fasting state into EDTA-coated tubes, immediately centrifuged at 4 °C (3000× *g* for 10 min), and plasma was aliquoted and stored at −80 °C until analysis.

### 2.4. Measurement of Plasma Resolvins

Plasma levels of resolvins D1 and D2 were quantified using the available enzyme-linked immunosorbent assay (ELISA) kits (Mybiosource, Inc., San Diego, CA, USA): Human Resolvin D1 (RvD1) (Cat. No: MBS053145, sensitivity 5.0 pg/mL) and Human Resolvin D2 (RvD2) (Cat. No.: MBS051498, sensitivity 5.0 pg/mL), according to the manufacturer’s protocol. Moreover, the manufacturer declares that no significant cross-reactivity or interference between RvD1, RvD2, and their analogues was observed when using these kits. Of note, although the manufacturer indicates no significant cross-reactivity problems, some degree of cross-reactivity/variability cannot be entirely ruled out due to the complexity of the molecular structures of lipid mediators in plasma.

The laboratory measurements of resolvins D1 and D2 were conducted in a blinded fashion. Laboratory personnel responsible for sample processing and quantification were not aware of group allocations (i.e., breast cancer subtype or control status).

### 2.5. Statistical Analyses and Sample Size Calculation

Continuous variables were described as means ± standard deviation or medians (interquartile range), as appropriate. Paired *t*-tests or Wilcoxon signed-rank tests were used to compare pre- and post-supplementation resolvin levels. Between-group comparisons were conducted using ANOVA or Kruskal–Wallis tests for normal or non-normally distributed data, as appropriate, and post hoc analysis was performed to assess differences between subgroups.

Based on previously published data on plasma DHA levels before and after oral DHA supplementation using the same formulation [8], we estimated the sample size required to detect a statistically significant difference in plasma resolvins levels—bioactive lipids directly derived from DHA metabolism—between breast cancer patients and healthy controls, using the changes in DHA plasma levels [8].

A one-tailed Wilcoxon–Mann–Whitney test was performed, with a significance level (α) of 0.05, 80% power, and an allocation ratio of 3:1 (patients to controls). Assuming an expected mean increase in DHA of 1.3 µg/mL in cancer patients and 1.07 µg/mL in controls, with an effect size = 1.15, the minimum required sample size was 21 breast cancer patients and 7 controls.

To account for an anticipated dropout rate of 15–20%, we enrolled 34 participants: 26 breast cancer patients and 8 healthy controls. Statistical significance was set at *p* < 0.05. Analyses were performed using IBM SPSS version [v28.0.1.1] and GPower 3.1 software for sample size calculation.

## 3. Results

### 3.1. Patient’s Characteristics at Baseline

We enrolled a total of thirty-four women; twenty-six were breast cancer patients with a mean age of 47 years ± 8.7, and eight served as controls, were affected by benign breast disease, and had a mean age of 47 years ± 5.4.

Among the breast cancer group, seven patients presented with sporadic breast cancer (sporadic group), nine with a positive familial history of breast cancer (familial group), and ten with a mutation in the BRCA 1/2 gene (mutated group). Other characteristics, including the immunohistochemical and the comorbidities, are summarized in Table 1.

### 3.2. Baseline Plasma Resolvin D1 and D2 Levels Among Breast Cancer Patients and Controls

**Figure 1:**
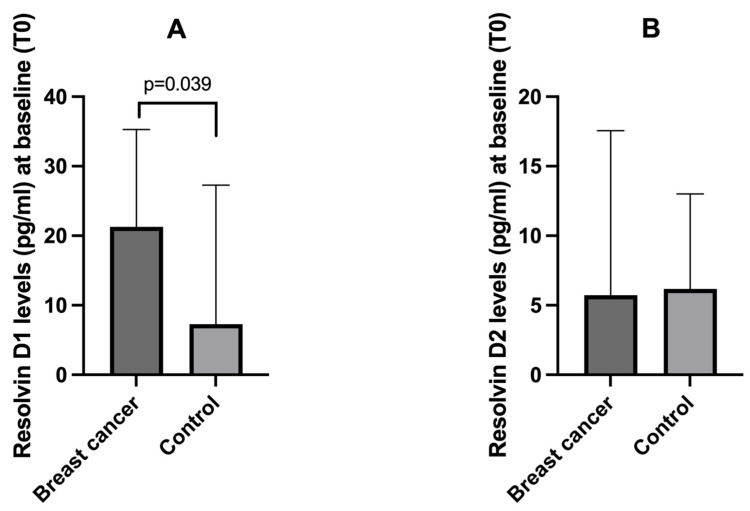
Baseline (T0) plasma levels of resolvin D1 (panel (**A**)) and resolvin D2 (panel (**B**)) in breast cancer patients and controls. Data are expressed as median with 95% CI.

Breast cancer patients showed higher plasma levels (pg/mL) of resolvin D1 compared to controls (median 21.3 vs. 7.3, respectively) (*p* = 0.039), whereas no difference was present between the two groups for resolvin D2 levels (Figure 1).

**Figure 2:**
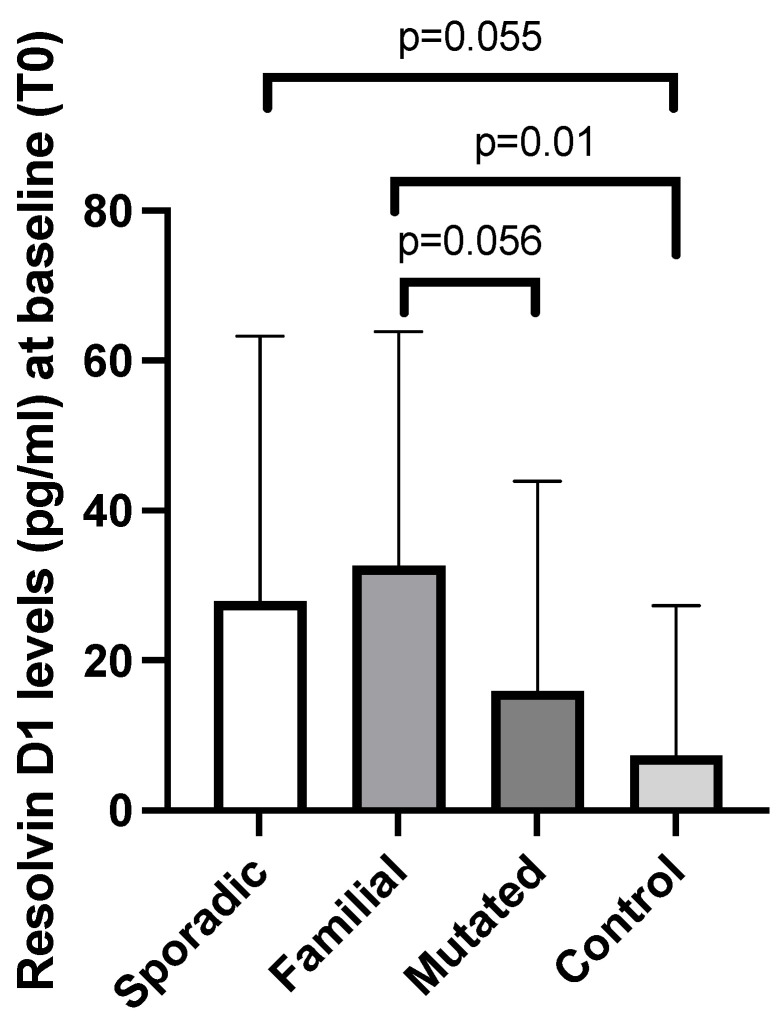
Baseline (T0) plasma levels of resolvin D1 in breast cancer patients according to the type of presentation (sporadic, familial, and BRCA1/2-mutated) and in controls. Data are expressed as median with 95% CI. *p*-Values are obtained from post-hoc analysis.

Considering the different forms of breast cancer presentation, patients in the familial group showed higher levels of resolvin D1 vs. controls (*p* = 0.01), and a similar behavior was observed vs. the mutated BRCA1/2 group (*p* = 0.056); this trend was present also for resolvin D1 when comparing the sporadic group vs. controls (*p* = 0.055) (Figure 2).

No differences were seen in resolvin D2 levels in the same comparisons. No significant difference in resolvin D1 and D2 levels was found according to high (≥20%) vs. low (<20%) Ki67 expression and according to the other immunohistochemical characteristics (luminal A, luminal B, HER2+, or triple-negative).

### 3.3. Changes from Baseline (T0) in Plasma Resolvin D1 and D2 Levels After DHA Oral Supplementation (T1) Between Patients and Controls

In both cancer patients and controls, we did not observe significant changes from baseline (T0) to the timepoint after DHA oral supplementation (T1) in the levels of resolvins D1 and D2 (Table 2).

**Figure 3:**
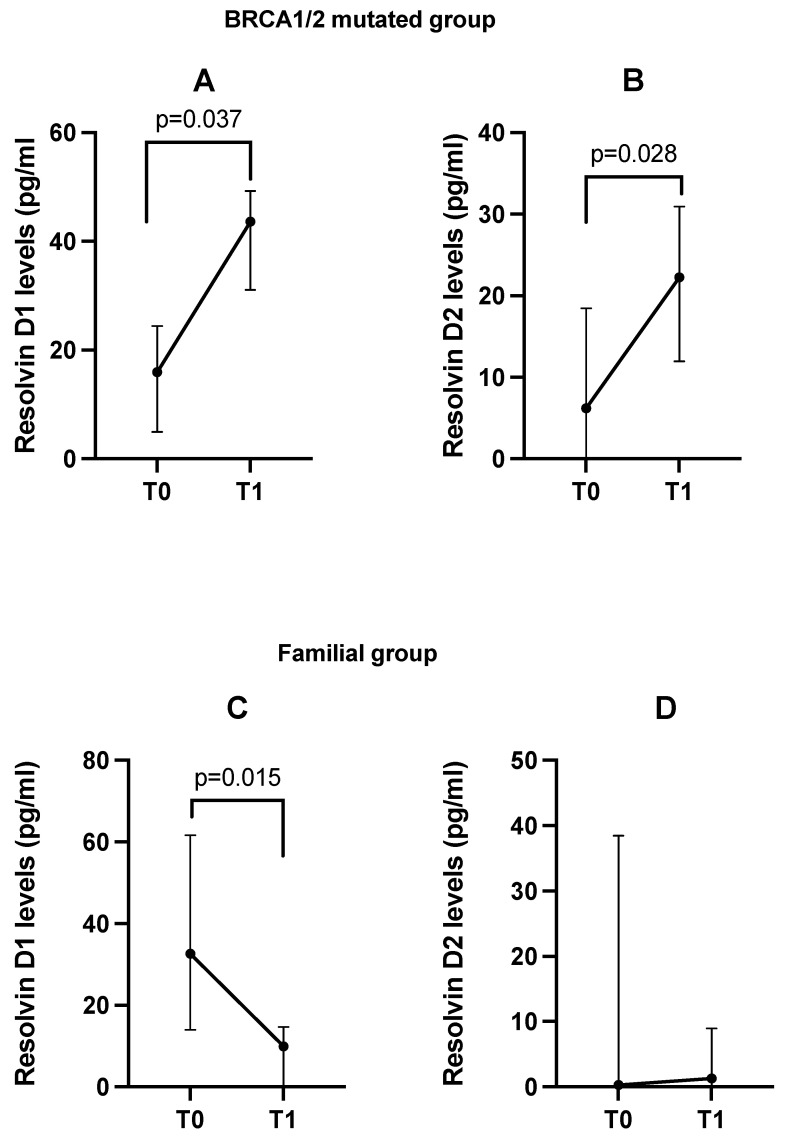
Change from baseline (T0) in plasma resolvin D1 and D2 levels after DHA supplementation (T1) in BRCA1/2-mutated (Panels (**A**,**B**)) and familial (Panels (**C**,**D**)) groups. Data are presented as median with IQR.

However, when stratifying according to the form of breast cancer presentation, we found in patients with mutated BRCA1/2 mutation a significant increase in the plasma levels of resolvins D1 and D2 (median Δ%_T0-T1_: +185.8 and +101.2, respectively) (*p* = 0.037 and *p* = 0.028). In parallel, in the familial group, we observed a decrease in resolvin D1 levels after DHA supplementation (median Δ%_T0-T1_: −73.6%, *p* = 0.015) (Figure 3).

**Figure 4:**
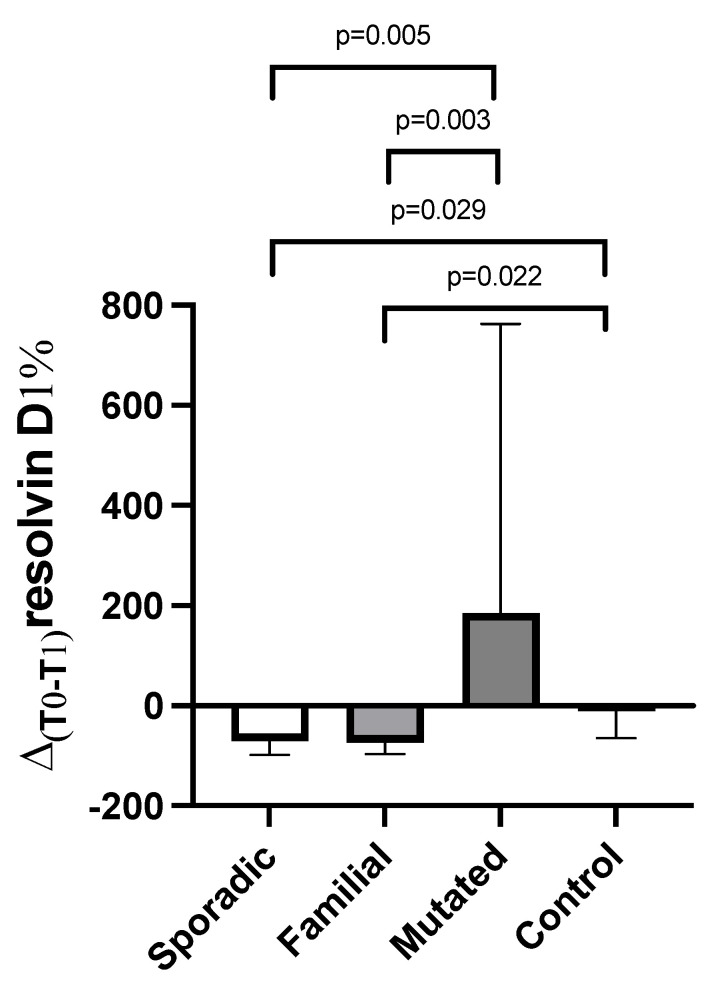
Change of plasma resolvin D1 levels (Δ%T0-T1) in breast cancer patients (according to the type of presentation) and in controls. Data are expressed as median with 95% CI. *p*-values are obtained from post-hoc analysis.

When considering the Δ%_T0-T1_ of resolvin D1 across the groups, patients in the mutated group presented higher Δ%_T0-T1_ of resolvin D1 vs. the familial and sporadic group, whereas a lower Δ%_T0-T1_ of resolvin D1 was found in the familial and sporadic groups vs. controls (Figure 4). No differences were present in the Δ%_T0-T1_ of resolvin D2 according to the different forms of breast cancer presentation.

**Figure 5:**
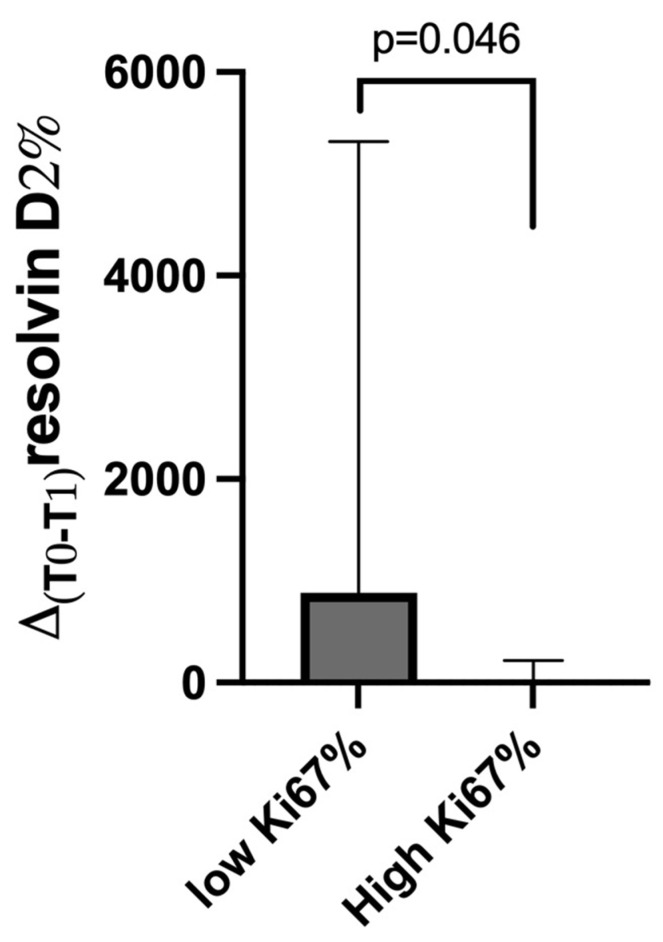
Change of plasma resolvin D2 levels (Δ%T0-T1) in breast cancer patients with high Ki67% (≥20) vs. low Ki67% (<20) levels. Data are expressed as median with IQR.

Taking into account the immunohistochemical characteristics, breast cancer patients with low Ki67 expression (<20%) showed higher Δ%_T0-T1_ of resolvin D2 compared to those with high Ki67 expression (≥20%) (Figure 5) (*p* = 0.046). No difference was found in resolvin D1 levels in the same comparison. No differences were also present according to other characteristics (luminal A, luminal B, HER2+, or triple-negative) for both D1 and D2 plasma levels.

## 4. Discussion

Our study includes novel findings on the plasma levels of resolvins D1 and D2 in breast cancer patients compared to controls and their modulation by DHA oral supplementation over time. These observations provide insights into the potential role of resolvins in the inflammatory environment of breast cancer and their interaction with tumor characteristics and potential therapeutic interventions.

In particular, at baseline (T0), resolvin D1 levels were significantly higher in breast cancer patients compared to controls, while no differences were observed for resolvin D2 levels. This suggests that resolvin D1, but not D2, may reflect an adaptive response to the pro-inflammatory state often associated with cancer disease. Elevated resolvin D1 in breast cancer could indicate an active but insufficient resolution of inflammation, which aligns with its role as a specialized pro-resolving lipid mediator [13]. The lack of difference in D2 levels might point to a more stable or less responsive role of this mediator in early inflammatory modulation in breast cancer.

Among the breast cancer subgroups, patients with BRCA1/2 mutation exhibited significantly lower baseline levels of resolvin D1 compared to other subgroups. This finding may indicate a reduced ability to counteract inflammation in this specific group, potentially contributing to worse outcomes [22]. Resolvins, particularly D1, are known for their anti-inflammatory and pro-resolving properties, and their deficiency could impair the resolution phase of inflammation, exacerbating the tumor microenvironment’s pro-inflammatory state [23]. However, in the present study, we do not have information in terms of outcomes, and longitudinal data are necessary to establish whether these low baseline levels correlate with worse prognosis or more aggressive disease progression in patients with BRCA1/2 mutations.

Interestingly, we observed that changes in resolvin D1 and D2 levels from T0 to T1 varied across subgroups. In the mutated BRCA1/2 group, we found a significant increase in both resolvins D1 and D2 concentrations. This could indicate an enhanced response to DHA supplementation in this group, potentially reflecting better incorporation or metabolism of DHA. Interestingly, the familial group demonstrated a significant reduction in resolvin D1 levels post-supplementation, suggesting a possible attenuation of anti-inflammatory/resolving properties in this specific setting. While this observation could indicate a dysregulated response to DHA supplementation, its clinical implications remain uncertain. In this light, familial cases may not benefit from enhanced enzymatic conversion of DHA to resolvins, possibly due to genetic, epigenetic, or microenvironmental differences that impact the biosynthesis of these mediators.

The significant increase in resolvin D1 levels in the BRCA1/2-mutated group following DHA supplementation is particularly notable. This could reflect a better incorporation of DHA into cell membranes or enhanced intestinal absorption of DHA in this group. Genetic or metabolic factors associated with BRCA1/2 mutations might contribute to increased ability to synthesize resolvins from DHA. This observation suggests a potential therapeutic window for DHA supplementation in these patients, as increased resolvin D1 levels could enhance anti-inflammatory and pro-resolving mechanisms in breast cancer.

Preclinical studies have demonstrated their potential anticancer properties. In particular, the study by Sulciner et al. [24] provided evidence that some resolvins, including RvD1 and RvD2, can inhibit debris-stimulated tumor growth by enhancing the clearance of cellular debris through macrophage-mediated phagocytosis. Additionally, in vitro studies have shown that RvD1 can reduce cancer growth by stimulating anti-cancer activities of polymorphonuclear neutrophils (PMNs), further supporting its role in tumor suppression [16]. Considering this evidence, our findings showing that DHA supplementation determines increased levels of RvD1 and RvD2 in specific subgroups—such as BRCA1/2-mutated patients—may reflect the activation of endogenous pro-resolving and anti-tumor pathways. Although our study was not designed to assess clinical outcomes, the observed resolvins modulation in this specific setting (breast cancer naïve to any anticancer therapy), driven by a specific metabolic/nutritional intervention (DHA supplementation), appears clinically meaningful. These results provide a rationale for future clinical studies aimed at evaluating the therapeutic relevance of resolvins in breast cancer and their potential integration into personalized metabolic and nutritional care strategies.

Moreover, the delta change in resolvin D2 levels (from T0 to T1) in patients with high Ki67 expression is noteworthy. Ki67 is a marker of tumor proliferation, and its high expression is often associated with aggressive cancer and worse prognosis [25,26,27]. The reduced delta change in resolvin D2 levels in this subgroup may indicate impaired resolution of inflammation, potentially contributing to the high proliferative state of these tumors. Alternatively, the aggressive nature of these cancers might suppress the synthesis or bioavailability of resolvin D2, further exacerbating the pro-inflammatory environment. It remains uncertain whether this is a cause or a consequence of increased tumor proliferation, but it indicates the importance of exploring the role of resolvins in cancer biology.

Our study has several limitations, including the lack of mechanistic insights into the observed variations in resolvin levels after DHA supplementation, particularly the reduction in resolvin D1 levels in the familial breast cancer group. While the increase in resolvin levels in the mutated group suggests enhanced incorporation or metabolism of DHA, the mechanisms underlying the reduction in some resolvins post-supplementation remain unclear, as do the effects on markers of systemic inflammation, which were not tested here. Factors such as genetic variability, differences in inflammatory status, metabolic pathways, or interactions with the tumor microenvironment may play a role.

In addition, regarding the methods used, as acknowledged by the manufacturer of the ELISA kits, given the limited data available in the literature, it cannot be ruled out that, due to the limitations of current skills and knowledge, it is impossible to completely assess cross-reactivity between RvD1 and all possible analogues; therefore, some degree of cross-reactivity may still exist. On the other hand, although LC-MS/MS remains the reference standard method, ELISA kits are widely used due to their lower cost and ease of implementation. Notably, RvD1 human plasma concentration has been reported to be approximately 30 pg/mL when measured by LC-MS [28,29], and in our study, the ELISA kits we used produced plasma resolvin levels comparable to those obtained via LC-MS. In addition, a previous validation study conducted in humans showed a strong correlation (r = 0.93) between ELISA and LC-MS/MS measurements for plasma RvD1 [30]. However, some degree of cross-reactivity or variability cannot be entirely ruled out due to the complexity of lipid mediators in plasma.

## 5. Conclusions

In conclusion, our results suggest distinct baseline and post-supplementation profiles of resolvins D1 and D2 in breast cancer patients, reflecting their potential as biomarkers of resolution of inflammation and response to DHA supplementation. The significant increase in resolvin D1 levels in the BRCA1/2-mutated group after supplementation highlights the potential utility of DHA in modulating inflammation in this subgroup. Conversely, the reduction in resolvin D1 in the familial group raises concerns about their anti-inflammatory capacity and calls for further research to elucidate the mechanisms and outcomes in this cohort. Finally, the association of lower resolvin D2 changes with high Ki67 expression suggests a potential link between inflammation resolution and tumor aggressiveness, emphasizing the need for future studies to explore these relationships and their therapeutic implications.

## Figures and Tables

**Table 1:** Participants’ characteristics.

Parameter	Breast Cancer Patients (N = 26)	Controls (N = 8)
Age, years	47.4 ± 8.7	47.3 ± 5.4
Actual weight, kg	64.3 ± 12.8	59.1 ± 7.4
Height, cm	164.0 ± 6.9	160.3 ± 2.6
BMI, kg/m^2^	23.9 ± 4.3	23.0 ± 2.2
*Type of disease presentation*		
Sporadic, *n* (%)	7 (26.9)	/
Familial, *n* (%)	9 (34.6)	/
Mutated, *n* (%)	10 (38.5)	/
*Immunohistochemistry*		
Luminal-A, *n* (%)	11 (42.3)	/
Luminal-B, *n* (%)	1 (3.8)	/
HER2+, *n* (%)	8 (30.8)	/
Triple-negative, *n* (%)	6 (23.1)	/
High Ki67% (≥20%)	20 (76.9)	/
*Main Comorbidities*		
Diabetes, *n* (%)	1 (3.8)	0 (0)
Dyslipidemia, *n* (%)	3 (11.5)	0 (0)

**Table 2:** Plasma resolvin D1 and D2 levels * in breast cancer patients and in controls at baseline (T0) and after DHA oral supplementation (T1).

	Resolvin D1		Resolvin D2	
	T0	T1	T0	T1
**Breast cancer**	21.3 (7.9; 41.4)	14.6 (0.0; 40.9)	5.3 (0.0; 22.1)	8.9 (0.0; 26.9)
**Controls**	7.3 (0.3; 23.8)	17.9 (0.8; 30.8)	6.2 (0.0; 10.7)	9.9 (0.0; 27.4)

* No significant changes were observed from T0 to T1 in resolvin D1 and D2 plasma levels in breast cancer patients and in controls. Data are presented as median (25th; 75th percentile).

## Data Availability

The raw datasets generated, used, and analyzed in the current study are available from the corresponding author upon reasonable request.
